# Headache, Opiate Use, and Prescribing Trends in Women With Idiopathic Intracranial Hypertension: A Population-Based Matched Cohort Study

**DOI:** 10.1212/WNL.0000000000201064

**Published:** 2022-11-01

**Authors:** Nicola Jaime Adderley, Anuradhaa Subramanian, Mary Perrins, Krishnarajah Nirantharakumar, Susan P. Mollan, Alexandra Jean Sinclair

**Affiliations:** From the Institute of Applied Health Research (N.J.A., A.S., M.P., K.N.), University of Birmingham; Centre for Endocrinology, Diabetes, and Metabolism (K.N.), Birmingham Health Partners; Health Data Research UK (K.N.), Birmingham; Birmingham Neuro-Ophthalmology (S.P.M.), Queen Elizabeth Hospital; Metabolic Neurology (A.J.S.), Institute of Metabolism and Systems Research, University of Birmingham; and Department of Neurology (A.J.S.), University Hospitals Birmingham, Queen Elizabeth Hospital, United Kingdom.

## Abstract

**Background and Objectives:**

Physician prescribing habits for opiates and headache therapies have not been previously evaluated in a large, matched cohort study in idiopathic intracranial hypertension (IIH). Our objective was to evaluate opiate and headache medication prescribing habits in women with IIH compared with matched women with migraine and population controls. We also investigated the occurrence of new onset headache in IIH compared with population controls.

**Methods:**

We performed a population-based matched, retrospective cohort study to explore headache outcomes. Cross-sectional analyses were used to describe medication prescribing patterns. We used data from IQVIA Medical Research Data, an anonymized, nationally representative primary care electronic medical record database in the United Kingdom, from January 1, 1995, to September 25, 2019. Women aged 16 years and older were eligible for inclusion. Women with IIH (exposure) were matched by age and body mass index with up to 10 control women without IIH but with migraine (migraine controls), and without IIH or migraine (population controls).

**Results:**

A total of 3,411 women with IIH, 13,966 migraine controls, and 33,495 population controls were included. The adjusted hazard ratio for new onset headache in IIH compared with population controls was 3.09 (95% CI 2.78–3.43). In the first year after diagnosis, 58% of women with IIH were prescribed acetazolamide and 20% topiramate. In total, 20% of women with IIH were prescribed opiates within the first year of their diagnosis, reducing to 17% after 6 years, compared with 8% and 11% among those with migraine, respectively. Twice as many women with IIH were prescribed opiates compared with migraine controls, and 3 times as many women with IIH were prescribed opiates compared with population controls. Women with IIH were also prescribed more headache preventative medications compared with migraine controls.

**Discussion:**

Women with IIH were more likely to be prescribed opiate and simple analgesics compared with both migraine and population controls. Women with IIH trialed more preventative medications over their disease course suggesting that headaches in IIH may be more refractory to treatment.

Idiopathic intracranial hypertension (IIH)^1^ is a relatively uncommon condition characterized by raised intracranial pressure and papilloedema with no direct identifiable cause.^2,3^ It has clear diagnostic criteria which include normal neuroimaging and venography (which may have stigmata of raised intracranial pressure only) and a lumbar puncture opening pressure >25 cm CSF.^4^ This secondary headache condition has an established association with obesity, and recent weight gain is a key risk factor for developing the disease.^5-7^ There is an increasing incidence and prevalence of IIH.^8-11^ Most of those with IIH have headaches at diagnosis, with up to 86% reporting daily headaches.^12-16^ It is of interest that a relatively high portion of those with IIH headache will have a preexisting headache history, up to 44% in one study^17^ and up to 68% in another.^16^ After resolution of the raised intracranial pressure, 63% of the patients with IIH go on to have a persistent post-IIH headache at 12 months after initial diagnosis.^18^

Disability in patients with IIH is predominantly driven by headache^19,20^; however, vision and conditions such as depression and anxiety are known to be contributing factors in IIH.^21^ There are no licensed therapies for IIH, and there remains a lack of dedicated abortive or preventative headache therapy in IIH.^4,12,22,23^ There have been no specific studies evaluating the use of acute pain relief in IIH headache, although a number of studies have documented a regular use of analgesic medications in up to half of the patients with IIH.^14,17,24^ Headache in IIH is most frequently migraine-like,^12-15^ and therefore, migraine therapeutics are often used.^16,22,24^ However, the choice of drugs used for IIH headaches is limited because of the need to avoid drugs that may cause weight gain or those that cause exacerbation of depression or fatigue.^4,24^ The use of calcitonin gene-related peptide (CGRP) monoclonal antibodies as a headache preventative strategy has recently been demonstrated to be of benefit in those with chronic IIH headaches.^14^ It is hypothesized that medicine prescribing habits in IIH are likely to be different compared with age-matched and sex-matched individuals with migraine.

Medication overuse headache is defined by the International Headache Society as headache occurring on 15 or more days per month as a consequence of regular overuse of acute or symptomatic headache medication (on 10 or more or 15 or more days per month, depending on the medication) for more than 3 months.^25^ In primary headache disorders such as migraine, medication overuse is commonly reported as between 25% and 50%, where it is known to significantly exacerbate the underlying headache morbidity.^26^ In episodic migraine, there is clear documentation of medication overuse, where 11% routinely used opiates and 6% used compounds with barbiturates, which increased to over a third in those with chronic migraine.^27^ Opiate use not only is a driver for medication overuse headache but also leads to numerous other negative sequalae, including insomnia, constipation, cardiac problems, and suicide.

Medication overuse has been documented in IIH, occurring in up to 23% at diagnosis^17^ and up to 35% of those with established disease.^16^ Up to 37% have been noted to use analgesics daily because their disease becomes more chronic.^17^ When the intracranial pressure has settled, evidenced by resolution of papilloedema, up to 48% have medication overuse documented.^14^ The use of opiates in patients with IIH has not been previously evaluated.

The aims of this study were to evaluate the prescribing of opiates, headache therapies, and IIH medications in primary care before and after a diagnosis of IIH compared with control women both with and without migraine and to investigate the hazard of new onset headache in women with IIH compared with women with neither IIH nor migraine.

## Methods

### Study Design

We performed an age-matched and body mass index (BMI)–matched retrospective cohort study using data from 1st January 1995 to 25th September 2019.

### Data Source

Data for this study were extracted from IQVIA Medical Research Data (IMRD-UK), which incorporates data from The Health Improvement Network (THIN), a national database of electronic primary care records, which is generalizable to the UK population.^28^ The database contains anonymized medical records of more than 15 million patients from 808 practices in the United Kingdom. It comprises coded information on patient demographics, symptoms and diagnoses, drug prescriptions, consultations, and diagnostic tests and their results. An IIH diagnosis necessitates a hospital encounter for diagnostic investigations in the United Kingdom, and the diagnosis is then reported to general practice or primary care physicians; this process increases the likelihood that a record of IIH diagnosis in the primary care electronic record is accurate. Studies using IMRD data for IIH analysis have previously been published.^10^ Data were extracted using Data Extraction for Epidemiological Research (DEXTER).^29^

### Population

To ensure high data quality, general practices were eligible for inclusion in the study from the latest of 12 months after the date from which they reported acceptable mortality recording rates^30^ and 12 months after the practice began using electronic medical records. Women registered with any of the eligible general practices for at least 365 days formed the source study population. Adult women aged 16 years and older were included.

#### Inclusion Criteria for Women With IIH (Exposed Group)

IIH diagnosis was ascertained by the presence of an IIH clinical (Read) code in the patient's medical records (eTable 1A, links.lww.com/WNL/C262). Read codes are a hierarchical system of coding symptoms and diagnoses used in UK primary care since 1985.^31^ Any IIH patients with a simultaneous clinical code for hydrocephalus or cerebral venous sinus thrombosis (eTables 1B and 1C) were excluded, in case they had been miscoded. For drug prescriptions, only women with incident IIH (newly diagnosed during the study period) and their corresponding matched controls were included in the analysis to capture drugs prescribed from the time of diagnosis. To assess rates of new onset headache and migraine (Read code lists in eTables 1D and 1E), women with prevalent and incident IIH and their corresponding population controls were included; in a sensitivity analysis, only women with incident IIH (and their corresponding controls) were included.

#### Inclusion Criteria for Migraine Controls (Unexposed)

For each incident patient with IIH, up to 10 controls with a migraine diagnosis,^32^ but no IIH, and matched using propensity scores based on index date, age, BMI category, Townsend deprivation quintile, ethnicity, and smoking status were randomly selected. Propensity score matching was used to maximize the number of migraine controls.

#### Inclusion Criteria for Population Controls (Unexposed)

For each patient with IIH, up to 10 population controls—women without a record of IIH and without a record of migraine—matched on the index date for age (±1 year) and BMI (±2 kg/m^2^), were randomly selected. Controls with a diagnosis of hydrocephalus or cerebral venous sinus thrombosis were excluded.

### Follow-up Period

For incident/newly diagnosed patients with IIH, index date was the date of diagnosis. For prevalent exposed/IIH patients with a preexisting diagnosis, the index date was 1 year after registration or 1 year after the date the practice became eligible to take part in the study, whichever was the latest, to ensure sufficient time for documentation of important baseline comorbidities. Population controls were assigned the same index date as their corresponding patient with IIH to mitigate immortal time bias.^33^ For migraine controls, index date was date of migraine diagnosis; index year was included among the propensity score matching variables.

All patients were followed-up from index date until the date of the earliest of the following endpoints: outcome, death, patient left the practice, practice ceased contributing to the database, or study end (September 25, 2019).

### Outcomes

#### Drug Prescriptions

Prescription of the following drugs in women with incident IIH and both control groups was explored: analgesics, migraine prevention therapies, and carbonic anhydrase inhibitors (eTable 2, links.lww.com/WNL/C262). In the United Kingdom, prescribing of migraine preventative medications is guided by the National Institute of Health and Clinical Excellence and other national body recommendations. There are also disease-specific recommendations for prescribing in IIH headache^4,22^; however, they were introduced at the end of our study period. The choice of medications to include in the analysis was based on UK guidance, and therefore, medications such as gepants, lisinopril, and memantine, which are either unavailable or not recommended in the United Kingdom, were not included. Prescriptions for botulinum toxin and CGRP monoclonal antibodies for migraine have been recent additions to the National Health Service prescribing formulary, CGRP therapy was not initiated until after the study period in the United Kingdom, and botulinum toxin therapy has been used for refractory migraine but not IIH headaches. Furthermore, these are prescribed within hospital care and are not recorded in IMRD/primary care data and, therefore, could not be explored in this analysis. Ditans have recently been approved by the Food and Drug Administration and were not included.

#### Headache and Migraine Outcomes

Outcomes were new onset headache and migraine. Outcomes were defined by a first record of a relevant clinical (Read) code which specified the headache or migraine diagnosis (eTables 1D and 1E, links.lww.com/WNL/C262). Patients with a record of the outcome at baseline were excluded. New onset headache and migraine were compared only with population controls because all women diagnosed with migraine would have a record of headache or migraine at index date.

### Analysis

#### Drug Prescriptions

A series of annual cross-sectional analyses were performed to calculate total counts and proportions of patients within each exposure group with a prescription record for each of the drug categories during the year before index date and during each year up to 6 years after index. Drugs were categorized into the following groups: analgesics: all acute analgesics, opiates, simple analgesics, and triptans; migraine preventative therapies: all, epilepsy drugs, tricyclics, beta blockers, candesartan, and pizotifen and methysergide; and medicines used to treat IIH (acetazolamide, diuretics, and topiramate). The proportion of women prescribed therapies in each of the drug categories was plotted over time, as a proportion of participants still being followed up at the given time point. Finally, the number of headache preventative medicine classes each participant had been trialed on at baseline and at 3 years was counted.

#### Headache and Migraine

Incidence rates for new onset headache and migraine per 1,000 person-years were calculated in women with a record of IIH (prevalent or incident) and in the population control group. Cox proportional hazards regression was used to calculate crude and adjusted hazard ratios (aHRs) and their corresponding 95% CIs for rates in women with IIH compared with population controls for both outcomes. Regression models were adjusted for age category, BMI category (categorized as underweight [<18.5 kg/m^2^], normal weight [18.5–25 kg/m^2^], overweight [25–30 kg/m^2^], and obese [>30 kg/m^2^]), Townsend deprivation quintile, smoking status (categorized as nonsmoker, current smoker, and ex-smoker), eating disorder, severe mental illness, back pain, osteoarthritis, rheumatoid arthritis, fibromyalgia, epilepsy, obstructive sleep apnea, and polycystic ovary syndrome, based on biological plausibility and report ed associations in the literature.^10,34-38^ Mental health conditions were considered as a potential confounder because drugs for mental health can also be prescribed for headache prophylaxis. The proportional hazards assumption was checked using the Schoenfeld residuals test.

Inclusion of individuals with a prevalent (preexisting) IIH diagnosis increases sample size but may result in survival bias because anyone with prevalent IIH who had also developed the outcome before study entry would be excluded from the analysis. A sensitivity analysis was therefore performed limiting the analysis to patients with incident IIH (newly diagnosed during the study period and followed up from the time of diagnosis) and their corresponding population controls to explore any effect of survival bias.

All analyses were performed in Stata IC version 16. Two-sided *p* values were obtained; *p* values <0.05 were considered statistically significant.

### Missing Data

Missing data for BMI, Townsend deprivation quintile, ethnicity, and smoking status were treated as a separate missing category in propensity score matching and the regression models, where relevant. Exposed patients with missing BMI were matched to population controls with missing BMI. The absence of a clinical code for a disease or of a prescription code for a given medication was taken to indicate the absence of the corresponding disease or prescription, respectively.

### Standard Protocol Approvals, Registrations, and Patient Consents

The use of IMRD-UK is approved by the UK Research Ethics Committee (Ref. No. 18/LO/0441); in accordance with this approval, the study protocol was reviewed and approved by an independent Scientific Review Committee (Ref. No. 18THIN070). IMRD-UK incorporates data from THIN, A Cegedim Database. Reference made to THIN is intended to be descriptive of the data asset licensed by IQVIA. This work used deidentified data provided by patients as a part of their routine primary care.

## Results

### Baseline Characteristics

A total of 3,411 women with IIH, 13,966 migraine controls, and 33,495 controls were included in the analysis. Baseline characteristics are presented in Table 1. The mean age in prevalent and incident IIH and population controls was 34 years; in incident only IIH and corresponding matched controls, the mean age was 32 years in all exposure groups. The mean (SD) BMI was 35.7 (8.1), 29.4 (6.5), and 35.1 (7.7) in the incident IIH, migraine control, and population control groups, respectively. Approximately a quarter of the participants were smokers. The proportion of participants with back pain, polycystic ovary syndrome, osteoarthritis, epilepsy, fibromyalgia, sleep apnea, and severe mental illness was higher in women with IIH compared with both control groups (Table 1).

**Table 1:**
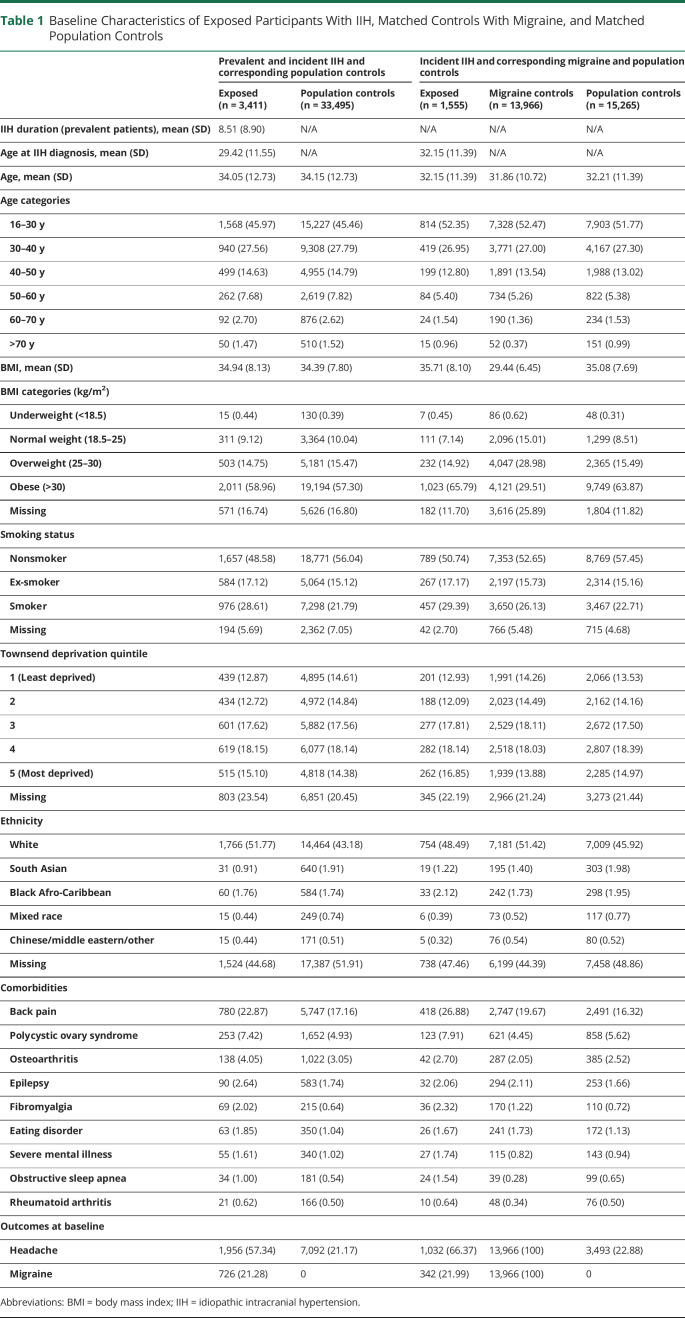
Baseline Characteristics of Exposed Participants With IIH, Matched Controls With Migraine, and Matched Population Controls

### Drug Prescriptions

#### IIH Treatments

As expected, acetazolamide was the most common drug prescribed in women with IIH within their first year of diagnosis (58%), followed by topiramate (20%) (eFigure 1, eTable 3, links.lww.com/WNL/C262). Both acetazolamide and topiramate continued to be prescribed, but in decreasing numbers, up to 6 years. Diuretics (furosemide and spironolactone) were rarely prescribed (eFigure 1).

#### Acute Analgesics

**Figure 1:**
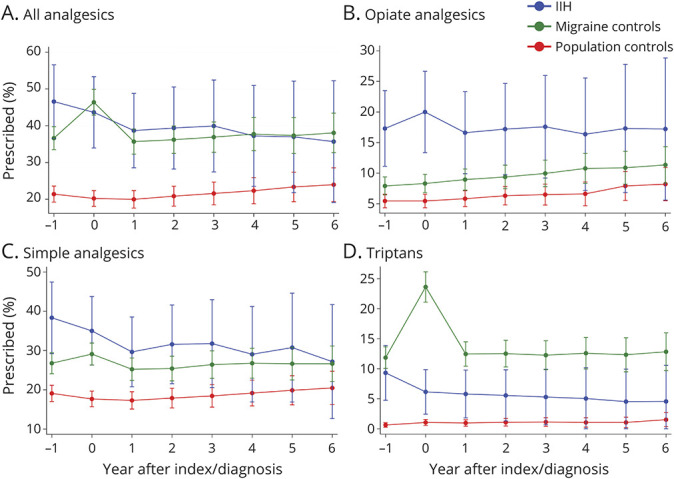
Analgesic Prescriptions in Women With IIH and Matched Migraine and Population Controls IIH = idiopathic intracranial hypertension.

Overall, prescribing of acute analgesics was similar in women with IIH and migraine controls, and lowest in population controls (Figure 1, eTable 4, links.lww.com/WNL/C262). A greater proportion of women with IIH were prescribed opiates compared with both migraine controls (approximately twice the proportion) and population controls (approximately 3-fold higher overall). The proportion of women with IIH prescribed analgesics remained relatively constant over time from the point of diagnosis (Figure 1).

#### Migraine Prevention Drugs

**Figure 2:**
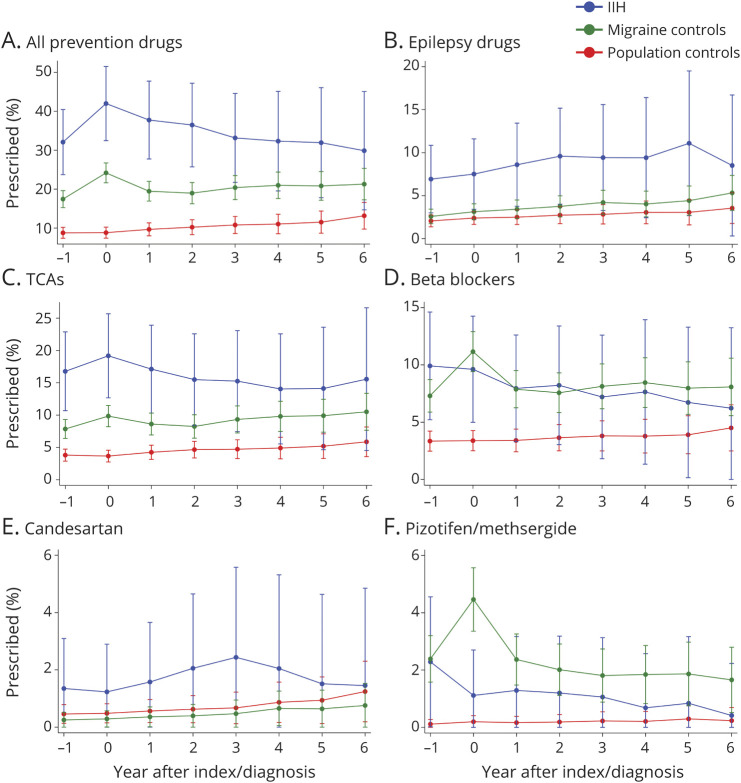
Preventative Prescriptions in Women With IIH and Matched Migraine and Population Controls IIH = idiopathic intracranial hypertension; TCA = tricyclic antidepressants.

**Figure 3:**
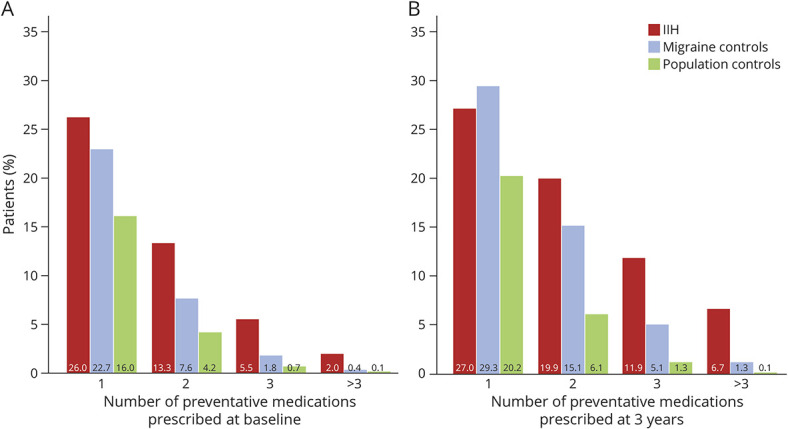
Number of Preventative Medications Initiated at Baseline and Within 3 Years in Women With IIH and Matched Migraine and Population Controls IIH = idiopathic intracranial hypertension.

Overall, prescribing of prevention drugs was substantially higher in women with IIH compared with either migraine controls (around 50% higher on average) or population controls (approximately 3-fold higher) (Figure 2, eTable 5, links.lww.com/WNL/C262). In particular, prescribing of epilepsy class drugs, tricyclic antidepressants, and candesartan was higher in women with IIH. Prescribing of pizotifen/methysergide was highest in migraine controls; prescribing of beta-blockers was similar in women with IIH and migraine controls, with around a 2- to 3-fold higher proportion prescribed beta-blockers compared with population controls. In women with IIH, for most of the preventative drugs, the overall trend was for prescribing to peak in the year of IIH diagnosis and then decline slightly thereafter (Figure 2). The number of preventative medications that had been prescribed was counted at index and 3 years. Those with IIH tried more preventative medications compared with the migraine and population controls (Figure 3). By 3 years, patients with IIH were more likely to have tried 2, 3, or more than 3 preventatives than the migraine controls.

### Headache and Migraine Outcomes (IIH and Population Controls Only)

A total of 1,455 women with IIH and 26,403 population controls (with neither IIH nor migraine) were included in the analysis for new onset headache. The crude incidence of new onset headache was 71.6 and 23.9 per 1,000 person-years in the IIH and population control groups, respectively (Table 2). Compared with control women, the aHR for new onset headache in women with IIH was 3.09 (95% CI 2.78–3.43). In a sensitivity analysis restricted to only women with incident IIH and their corresponding controls, aHR was 4.92 (95% CI 4.21–5.74) (eTable 6, links.lww.com/WNL/C262).

**Table 2:**
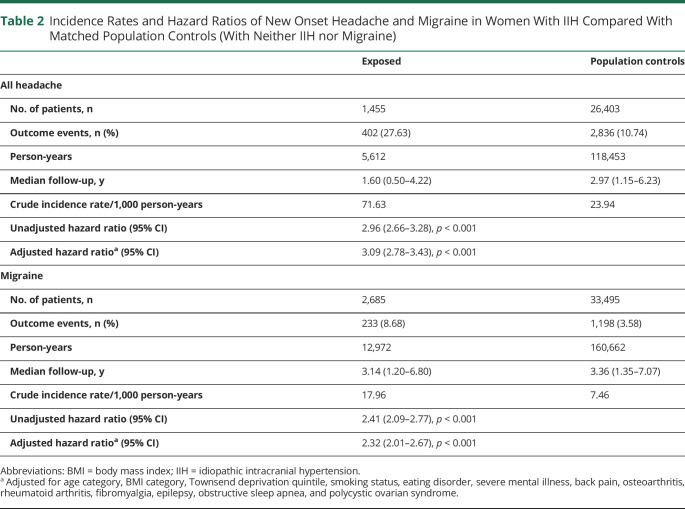
Incidence Rates and Hazard Ratios of New Onset Headache and Migraine in Women With IIH Compared With Matched Population Controls (With Neither IIH nor Migraine)

A total of 2,685 women with IIH and 33,495 population controls were included in the analysis for a diagnosis of new onset migraine. The crude incidence of migraine was 18.0 and 7.5 per 1,000 person-years in the IIH and population control groups, respectively (Table 2). Compared with population controls, the aHR for new onset migraine in women with IIH was 2.32 (95% CI 2.01–2.67). In a sensitivity analysis restricted to only women with incident IIH and their corresponding controls, aHR was 3.25 (95% CI 2.69–3.91) (eTable 6, links.lww.com/WNL/C262).

## Discussion

In this large population-based, age-matched, and BMI-matched cohort study, we found that women with IIH had a 3-fold increased hazard of new onset headache and more than twice the hazard of a diagnosis of new onset migraine compared with women without IIH (population controls). Prescribing data suggest that headache in IIH is challenging to treat, as evidenced by the increased opiate, simple analgesics, and preventative medications prescribed in IIH compared with the migraine and population control groups.

Although IIH has high headache morbidity,^1,14,17,20^ there has only been 1 open label study specifically evaluating headache treatment in IIH.^14^ When a priority setting partnership was concluded, understanding headache and new treatments was highly prioritized for the disease.^39^ This burden of headache is reflected in the prescribing patterns recorded here. The proportion of women with IIH prescribed simple analgesics remained at a relatively constant level over the study period from 1 year before diagnosis up to year 6 and was higher (ranging between 29% and 39%) than both control groups. A greater proportion of the IIH group were prescribed opiates compared with both migraine controls (approximately double) or population controls (approximately triple). This implies that headache in IIH remains uncontrolled and that the IIH population is at significant risk of medication overuse headaches. In other headache disorders, overuse of analgesic medications is believed to contribute to the frequency and severity of headache and may have a role in progression from episodic to chronic headaches.^40^ It is also notable that obesity and female sex are important risk factors for medication overuse, both of which are common characteristics of IIH.^41^

This study has demonstrated that existing migraine preventative drugs are commonly prescribed in IIH. These are currently used off-label without evidence of efficacy in this population. A number of the classes of headache prevention drugs can cause weight gain as an unwanted side effect. This limits treatment choices in IIH where weight loss is central to management and for remission of the disease.^4,7,22,42^ In this study, we have provided evidence of the prescribing of headache preventatives that could induce weight gain, and physicians should consider alternative options. Currently, as there are no licensed treatment options, the IIH population remains at significant risk of medication overuse.

A recent open label study using erenumab in 55 IIH women, with a mean disease duration of 10 years, recorded on average 3.7 preventative treatment failures,^14^ indicating headache in IIH is resistant to medical therapy used in migraine. The burden of headache seen in this small prospective study is replicated here. For escalation to second-line treatment of either botulinum toxin or CGRP monoclonal antibodies, the person has to fulfil a diagnosis of chronic migraine and importantly have trialed and failed at least 3 preventative drug treatments. To understand the proportion of people with migraine and those with IIH that fulfil the criteria for chronic migraine headaches who may be eligible for this escalation of treatment, the number of preventative medications that had been prescribed was measured both at diagnosis and 3 years. Those with IIH had trialed more classes of preventative medications, compared with both the migraine and population control groups. By 3 years, patients with IIH were more likely to have trialed 2, 3, or more than 3 preventative medicine classes than the migraine controls. These data further suggest that a persistent IIH headache is refractory to conventional migraine preventative treatment.^12^

Our research has several strengths. A large sample size has been used to explore a rare condition (IIH) and to understand its association with headache and migraine outcomes, and prescribing habits. Patients included in IMRD-UK are generalizable to the UK population. In the United Kingdom, diagnosis of IIH is made in the hospital setting in line with diagnostic criteria; however, the diagnostic criteria for IIH have evolved over time.^4,22,23^ The diagnosis is then communicated to the general practice. However, there is a known risk of diagnostic error causing misclassification bias^43^ and a possibility of data entry error in the hospital or general practice. To mitigate this, we excluded those with a record of hydrocephalus or cerebral venous sinus thrombosis. It is possible that there may be underrecording of IIH due to either undiagnosed cases or cases being diagnosed in secondary care and not being documented/recorded in the patient's primary care record. Overdiagnosis is possible but unlikely due to diagnosis being made by specialists in secondary care. Diagnosis of migraine in primary care has been found to be 98% accurate in accordance with the International Headache Society classification of migraine^44^; however, it is not possible to ascertain from the data whether some of those with new onset headache may have a migraine phenotype, and there is ongoing debate as to whether people with IIH have a chronic headache that is physiologically different from chronic migraine. Drug prescriptions are accurately recorded in primary care data; however, the use of paracetamol, aspirin, and nonsteroidal anti-inflammatory drugs obtained personally/over-the-counter by the patient could not be captured here; furthermore, any drugs prescribed in secondary care would not be captured in the data set. It is possible that the lower prescription rates of migraine preventatives observed in women with migraine compared with those with IIH may be partially due to undertreatment of migraine. We were not able to perform a sensitivity analysis in acute vs chronic IIH because information regarding papilloedema status is not recorded within the IMRD database. This may have clear influence on prescribing habits; however, overall, we note from the data that prescribing of analgesics continues long term (with the proportion remaining similarly high at least up to 5 years after diagnosis) in those diagnosed with IIH. The results detailing opiate prescribing would be representative of other international populations where opiate prescribing is permitted for pain and headache, but not necessarily reflect prescribing habits where opiate prescribing has been restricted by public policy or laws. As this study included women only, the findings are not directly generalizable to men with IIH.

This was the largest national study providing epidemiologic data evaluating prescribing habits for those with IIH and comparing them with matched migraine controls and population controls. The finding that opiate use in IIH was 3 times higher than in the population controls and double that observed in migraine controls is a major concern. There are likely to be multiple contributing factors, including the major burden of headache these patients experience. However, the consequences of such dominant opiate use in IIH are likely be extensive and contribute to the poor quality of life that has previously been noted. We also observed increased prescribing of simple analgesics and preventative drugs in IIH compared with migraine controls. These data may point toward a refractory nature of IIH headache. Headache management in IIH remains an unmet clinical need, and the development of targeted therapies may help reduce the multiple prescriptions of preventative migraine medications and curb the opiate prescribing trends.

## Glossary

BMI: body mass index
CGRP: calcitonin gene-related peptide
IIH: idiopathic intracranial hypertension
IMRD: IQVIA Medical Research Data
THIN: The Health Improvement Network
